# Clinic Atlanta Medical College

**Published:** 1873-12

**Authors:** E. A. Cobleigh, W. F. Westmoreland

**Affiliations:** Reporter; Professor of Surgery


### Clinic Atlanta Medical College.

E. A. COBLEIGH, Reporter.

W. F. WESTMORELAND, M.D., PROFESSOR OF SURGERY.

Case 1.—Wm. G., white, medical student. November 1st—Has secondary hemorrhage from extraction of a tooth eight days ago. Muriated tincture iron was applied to cavity after carefully removing all coagulated blood, and proved effectual in putting an end at once to the hemorrhage.

Case 2.—November 1st—Julia S., colored, æt. thirty-seven years, widow. Has troublesome excoriation in and around anterior nares, of three months' duration, as a consequence of secretions from the nose. Diagnosis—Eczema. Treatment—Cleanse the part daily with tepid water, and apply the following wash:

| | |
|---|---|
| ℞.—Zinci Sulph., . . . . . . | grs. v. |
| Plumbi Acetat, . . . . | grs. x. |
| Tr. Catechu, . . . . . . | f℥j. |
| Tr. Opii, . . . . . . . | f℥j. |
| Aquæ Pur., . . . . . . | f℥iij. |
| Misce. | |

After using the foregoing, sprinkle calamina preparata on the raw surface to prevent acrid secretions from flowing over sound skin adjacent to the affected tissues. Under this treatment the patient's disease was cured in about three weeks. She then complained of a new trouble, and, on examination an ulcerous sore was discovered over tibia of right leg. No indurated cervical glands were found, but, from history of case, a syphilitic taint of system was suspected. She was put upon the following:

| | |
|---|---|
| ℞.—Tr. Stillingiæ, . . . . . . | f℥ij. |
| Tr. Gentianæ, . . . . . . | f℥ij. |
| Potass. Iod., . . . . . . | ℥j. |
| Syrupi Simp., . . . . . . | f℥ij. |
| Aquæ Destil., . . . . . . | f℥ij. |

Misce. Sig.—One tablespoonful in full glass of water ter in die.

November 26th—Has been slowly improving, but complains that of late her medicine makes her sick. Has not been using enough water with it to properly dilute the dose. Ordered to persevere in same course of treatment, using more water, and gradually increase dose to two tablespoonfuls ter in die in two tumblerfulls of fluid.

Case 3.—October 25th, this patient, Mary T., æt. twenty-four years, colored, presented herself to be cured of an eruption covering, more or less thickly, the entire body, but being most profuse on face, breast and arms. The affection was an interesting one, from its rarity, and consisted in a simple obstruction of the outlets of thousands of sebaceous glands, causing them to retain their secretion and enlarge, thus becoming prominent elevations on cuticle. Puncturing these different-sized tumors, allowed the escape of sebaceous matter in various degrees of fluidity. No treatment instituted, except ordering the patient to pay strict attention to personal cleanliness. Disease has been in progress three years, but gives no troublesome symptoms, and annoys her only by reason of its disfiguring the face, which, by the way, is covered with old small-pox pits.

Case 4.—October 25th—Jane T., mother of the last patient, forty years old, colored, washerwoman. Has eczema on hands and shoulders of six weeks' standing, accompanied with much heat and itching, with watery discharge. Treatment—Wash affected parts with saturated solution of bicarbonate of soda several times a day, and take teaspoonful of soda in powder every two hours. November 1st—Is slowly improving. In addition to the other remedies, was told to powder the arms, after using the soda solution, with calamina preparata. November 26th—Nearly well. Wishing to test the unguentum diacyli hebræ, which is highly recommended in cases of this kind, the soda wash was discontinued on hands, and the ointment substituted. December 3d—Hands very much worse under diacylon treatment, and she was, therefore, put again on the alkalies. December 13th—Has continued to improve under alkaline treatment up to present time.

Case 5.—November 5th.—George W., white, æt. twenty-three years; laborer, traveling with a circus. Complains of having bloated very rapidly during the past week. Has been much exposed recently and for a long time. Had gonorrhœa two years ago. Privates badly swollen. Urine tested for albumen, and it was found to be quite abundant therein. Diagnosis: Acute albuminuria, caused by the exposure. Treatment: Chloroform in doses of fifteen drops every two hours. Patient discharged entirely cured on the 12th of November, the bloat having all disappeared.

Case 7—November 5th. C. D., negro, twenty-five years of age, shoemaker. Toes of right foot were amputated in 1868, and now has ulcer in the cicatrix of long duration. Another amputation must be performed to secure a good, flat and serviceable stump. November 19th, returned for amputation. Chloroform was administered and Dr. Westmoreland performed Pirogoff's operation. Patient recovered rapidly, no untoward symptoms manifesting themselves during cure.

Case 8.—'Cajah G., colored, twenty-six years of age, common laborer. This man came before the class November 8th. He has been confined to his bed for seven months; is fearfully emaciated and too weak to move unassisted. Had a fall one month before he took to his bed, and went home thereafter with a very severe pain in left side. One week later he passed bloody urine, which condition of things continued for several weeks, then ceased spontaneously. Complains of pain in left thigh, of five month's duration. Examination discovers the thigh twice as large as its fellow, and there are signs of fluctuation in tumefied part. On puncturing the tumor a small quantity of bloody pus escaped, but not much. The patient's position was changed and another opening made, when the pus flowed forth in abundance. Part was poulticed, patient sent to his home, and put upon a tonic course of treatment, with opiates. November 12th. Rested well after operation, but grew worse next day, there being now considerable pain, nervous excitement and prostration. Pulse about 115, and abscess discharging copiously, but appetite good and bowels in normal state of activity. Put upon brandy and quinine three times a day. Tr. Gentianæ f℥ij, Spts. Frumenti, Oiss, Quiniæ Sulph. gr. lij; two tablespoonfuls to be taken at a dose. November 14th. Pulse reduced to 90, and general improvement of symptoms. December 13th. Patient's condition has been fluctuating between improvements and relapses, but he is now doing well and seems in a fair way to recover under the treatment last instituted, which has been, and will be, continued until he is perfectly convalescent.

CASE 12—November 15th. Mrs. D., white, thirty-eight years of age, widow, four children, a housewife. This is a case of rupia of tertiary syphilis. Patient was before the class last year, before her disease had progressed to its present state, was put upon treatment and got along nicely but thought herself nearly well and ceased attending our Clinics. She now has some sore throat and pain in left arm. On examination a number of ulcers and abscesses were found on the arm and shoulder and in axilla of left side. Also a few old scars on trunk, showing where other abscesses have broken and healed.

| | | | |
|---|---|---|---|
| ℞.—Potass. Iodidi, | . . . . . . | | ℥iss. |
| Tr. Gentianæ, | . . . . . . | | |
| Tr. Stillingiæ, | . . . . . | aa | f. ℥ij. |
| Aquæ, | . . . . . . . . | | f. ℥iv. |

Misce. Take tablespoonful of this ter in die in full glass of water and gradually increase the dose until double the quantity of both medicine and water are used. December 10th. Is improving. Several abscesses have been punctured and these, with some of the ulcers, are healing. Continue the former treatment.

CASE 15—Miss DeL., æt. twenty years, white. Came before the class on November 15th, with a large sebaceous cyst behind right ear. Expressing herself ready for an operation. Dr. Westmoreland proceeded to dissect out the cyst entire. The incision was then closed with silver suture and the patient discharged.

CASE 17—November 22d, Emma L., æt. thirty-six years, white, married. Has a tumor on anterior surface of the neck. Diagnosis, bronchocele. This patient will be put upon treatment with the galvanic battery at an early date, to test the efficacy of that means of discussing tumors of this kind; nothing done for her at present.

CASE 21—November 26th. J. H. S., twenty-six years of age, white, a railroad operative. Right leg was amputated last March, half-way between the knee and ankle. Now has ulcer in the cicatrix and complains of terrible pain in the whole stump, with much constitutional disturbance. Patient was anæsthetized and Professor W. proceeded to perform a second operation, taking off the stump about four inches above its extremity. After amputation it was found that osteo-myelitis of the tibia set up after the first operation, had caused his pain and constitutional trouble, there being a large quantity of pus in the portion of that bone which the doctor cut off. December 8th—patient doing well, and the stump is healing very rapidly. Took the cars for his home to-day.

Case 22.—James S., æt. eighteen years, negro, laborer. This boy came to clinic November 29th, with phymosis from gonorrhœa. By forcible dilatation the phymosis was overcome, and patient was put upon proper remedies to cure his gonorrhœa.

Case 23—December 3d, Warren J., shoemaker, colored, twenty-four years of age. Patient has an abundant crop of syphilitic warts covering glans, prepuce, frænum and part of the cuticle of his penis. These were treated by clipping them off with scissors. Nothing done for his syphilis, as he is under another physician's care for that.

Case 26—December 3d, Martin N., white, child of sixteen months. The child's father brings him here to be cured of a deformity. Examination shows us that this is a case of talipes varus. Dr. W. immediately operated, making sub-cutaneous section of tendo Achilis, after which the shoe for the cure of club-foot was applied.

Case 30—December 10th, Chas. M., child eight months old, white. This little patient was brought before the class for same trouble as case 26, and was treated in same manner, save that no section of the tendon, only a division of the plantar fascia, was necessary.

